# Scalable manufacturing platform for the production of methemoglobin as a non-oxygen carrying control material in studies of cell-free hemoglobin solutions

**DOI:** 10.1371/journal.pone.0263782

**Published:** 2022-02-16

**Authors:** Xiangming Gu, Richard Hickey, Antara Rath, Andre F. Palmer

**Affiliations:** William G. Lowrie Department of Chemical and Biomolecular Engineering, The Ohio State University, Columbus, OH, United States of America; University of Newcastle, AUSTRALIA

## Abstract

Methemoglobin (metHb) arises from the oxidation of ferrous hemoglobin (HbFe^2+^, Hb) to ferric hemoglobin (HbFe^3+^, metHb), which is unable to bind gaseous ligands such as oxygen (O_2_) and carbon monoxide (CO), and binds to nitric oxide (NO) significantly slower compared to Hb. Therefore, metHb does not elicit vasoconstriction and systemic hypertension *in vivo* due to its extremely slow NO scavenging rate in comparison to cell-free Hb, but will induce oxidative tissue injury, demonstrating the potential of using metHb as a control material when studying the toxicity of cell-free Hb. Hence, the goal of this work was to develop a novel manufacturing strategy for production of metHb that is amenable to scale-up. In this study, small scale (e.g. 1 mL reaction volume) screening experiments were initially conducted to determine the optimal molar ratio of Hb to the oxidization agents hydrogen peroxide (H_2_O_2_) or sodium nitrite (NaNO_2_) to achieve the highest conversion of Hb into metHb. A spectral deconvolution program was employed to determine the molar fraction of various species (hemichrome, metHb, oxyHb, metHb-${NO}_{2}^{-}$, and NaNO_2_) in solution during the oxidation reaction. From this analysis, either a 1:1 or 1:5 molar ratio was identified as optimal molar ratios of Hb:NaNO_2_ (heme basis) that yielded the highest conversion of Hb into metHb with negligible amounts of side products. Hence in order to reduce the reaction time, a 1:5 molar ratio was chosen for large scale (i.e. 1.5 L reaction volume) synthesis of bovine metHb (metbHb) and human metHb (methHb). The biophysical properties of metHb were then characterized to elucidate the potential of using the synthesized metHb as a non-O_2_ carrying control material. The haptoglobin binding kinetics of metHb were found to be similar to Hb. Additionally, the synthesized metHb was stable in phosphate buffered saline (PBS, 50 mM, pH 7.4) at 4°C for approximately one week, indicating the high stability of the material.

## 1 Introduction

*In vivo* exposure to cell-free hemoglobin (Hb) results in adverse side-effects [1, 2]. For experimental studies investigating the toxicity of cell-free Hb, crystalloid solutions such as lactated Ringer’s (LR) have been used as the standard control to discern the effects of Hb on physiological responses associated with vasoactivity and oxidative stress [3–5]. Unfortunately, LR is not an ideal control material when studying the toxicity of Hb, since it does not possess the same structure or equivalent half-life of cell-free Hb [6, 7]. Fortunately, Hb can be converted to methemoglobin (metHb), in which the iron atom is oxidized to the non-functional ferric state (Fe^3+^) from the functional ferrous state (Fe^2+^). Since metHb (HbFe^3+^) is an oxidized form of native Hb (HbFe^2+^), it cannot bind and release gaseous ligands such as oxygen (O_2_) and carbon monoxide (CO) [8, 9]. Although metHb does bind to nitric oxide (NO), the NO binding rate of metHb is significantly slower than the reaction between NO and native Hb *in vitro*, which does not elicit vasoconstriction and systemic hypertension *in vivo* [10–12]. Additionally, metHb can be removed from the systemic circulation via haptoglobin (Hp) binding and subsequent CD163 receptor mediated endocytosis as well as via elimination through the kidneys and thus most likely possesses a similar half-life to Hb [13]. Previously, it was observed in a canine model that the infusion of metHb did not increase mean arterial pressure (MAP) in comparison to cell-free Hb, demonstrating the inability of it to participate in NO signaling pathways [14]. Unlike cell-free Hb which can scavenge NO and subsequently elicit vasoconstriction and systemic hypertension, metHb does not elicit those side-effects. Hence, metHb can function as a suitable non-oxygen carrying control material when studying the vasoactive toxicity of cell-free Hb. Very recently, metHb was used as a precursor to synthesize liposome-encapsulated metHb nanoparticles as an antidote to treat cyanide poisoning [15, 16].

MetHb is typically prepared by reacting excess potassium ferricyanide (K_3_Fe(CN)_6_) (on a per heme basis) with Hb [17, 18]. The reaction mixture is then separated on a desalting column to remove ${Fe(CN)}_{6}^{3-}$ and ${Fe(CN)}_{6}^{4-}$ from metHb. However, the chromatographic desalting process is not the best approach for bench-top or pilot scale purification of metHb due to sample dilution during the purification process.

In this study, to synthesize pure metHb, either sodium nitrite (NaNO_2_) or hydrogen peroxide (H_2_O_2_) was used to oxidize Hb, since both molecules are reported as strong oxidizing agents and are the most commonly used Hb oxidizing agents in the literature [19, 20]. Various molar ratios of Hb:oxidization agent were screened in small scale reaction volumes (1 mL) to determine the optimal type of oxidization agent and molar ratio of Hb:oxidization agent that yielded the highest conversion of Hb into metHb. To monitor the oxidation of Hb during the reaction, a spectral deconvolution program (Alchromy) was employed to determine the molar fraction of various species (hemichrome, metHb, oxyHb, metHb-${NO}_{2}^{-}$, and NaNO_2_) in solution based on the standard UV-visible spectra of each pure species. From this analysis, we identified an optimal molar ratio of Hb:NaNO_2_ that yielded the highest conversion of Hb into metHb with negligible levels of impurities among samples oxidized with either NaNO_2_ or H_2_O_2_ at different molar ratios.

To scale-up production of metHb, a novel synthesis and purification protocol was developed in this study. In the metHb synthesis protocol, NaNO_2_ was injected into a well-mixed batch reactor containing bovine Hb (bHb) or human Hb (hHb). After the reaction went to completion, the synthesized metHb was then subject to diafiltration with LR solution until ~250 mL of the product was obtained at a total protein concentration of ~100 mg/mL and a percentage of metHb (metHb level) of 99.99%. The biophysical properties of metHb were then characterized to elucidate the potential of using it as a non-O_2_ carrying control material. At the end of the tangential flow filtration (TFF) purification process, only metHb was present in solution with undetectable levels of nitrite. Each batch of metHb was characterized via spectral deconvolution analysis, Hp binding kinetics analysis, size exclusion HPLC (SEC-HPLC) analysis, and subjected to storage stability analysis.

## 2 Materials and methods

### 2.1 Materials

Sodium citrate anticoagulated whole bovine blood was purchased from Quad Five (Ryegate, MT). Expired packed human red blood cell (RBC) units were obtained from Transfusion Services (Wexner Medical Center, The Ohio State University, Columbus, Ohio). NaNO_2_, H_2_O_2_) calcium chloride (CaCl_2_·2H_2_O), sodium phosphate monobasic (NaH_2_PO_4_), sodium phosphate dibasic (Na_2_HPO_4_), potassium chloride (KCl), sodium hydroxide (NaOH), sodium chloride (NaCl), and sodium lactate were purchased from Sigma-Aldrich (St. Louis, MO). Hollow fiber (HF) modules with 500 kDa and 50 kDa molecular weight cut off (MWCO) were procured from Repligen (Rancho Dominguez, CA). K_3_Fe(CN)_6_ and potassium cyanide (KCN) were obtained from Fisher Scientific (Pittsburgh, PA). 0.2 μm syringe filters were purchased from Thermo Fisher Scientific (Waltham, MA).

### 2.2 Hb purification

Bovine/human red blood cells (RBCs) were washed via centrifugation with 0.9% saline and lysed with phosphate buffer (PB) (3.75 mM, pH 7.4). Tangential flow filtration (TFF) HF modules with MWCOs of 500 kDa and 50 kDa were then used to purify and concentrate bHb/hHb as described in the literature [21].

### 2.3 Small scale optimization of Hb:oxidization agent stoichiometry

A small scale (1 mL) metHb synthesis study using two different types of oxidization agents was conducted to determine the optimal dose of oxidizing agent needed to fully convert Hb into metHb. In this study, hydrogen peroxide (H_2_O_2_) and sodium nitrite (NaNO_2_) were prepared at 1:1, 1:5, and 1:10 molar ratio of Hb:oxidization agent within 1 mL UV-visible quartz cuvettes. bHb/hHb was diluted to 20 mg/mL using phosphate buffered saline (PBS, 0.1 M, pH 7.4). Stock solutions of NaNO_2_ and H_2_O_2_ were prepared at 8.5 mg/mL and 4.2 mg/mL, respectively. To obtain the 1:1, 1:5, and 1:10 molar ratio of Hb:oxidization agent solution, 1 mL bHb/hHb solution was mixed with 10/50/100 μL of the NaNO_2_/H_2_O_2_ stock solution, respectively. The absorbance spectra from 350−700 nm was simultaneously monitored for 21 hours at room temperature in 6 parafilm-sealed quartz cuvettes and a blank quartz cuvette with PBS (0.1 M, pH 7.4) using an HP 8452A diode array UV-visible spectrometer (Olis, Bogart, GA).

To determine the fraction of multiple Hb species in solution, we previously developed a spectral deconvolution program (Alchromy) based on the extinction coefficients and absorbance spectra of each pure species. For the reaction between bHb/hHb and NaNO_2_, the fractional composition of hemichrome, oxyHb, metHb, ferrylHb, metHb-NO_2_^-^, and NaNO_2_ in solution was calculated at each time point during the 21 hour reaction time period using the Alchromy program. Similarly, for the reaction between bHb/hHb and H_2_O_2_, the fractional composition of hemichrome, oxyHb, and metHb was calculated as described above.

### 2.4 UV-visible spectra deconvolution analysis

Samples containing Hb were analyzed via UV-visible spectroscopy and compared to the UV-visible spectra of pure species of Hb bound to various ligands through spectral deconvolution. The open-source Python package Alchromy (www.alchromy.com) was used to interpret and analyze spectra, and leveraged a nonlinear least squares fitting function of the SciPy package to determine the fraction of various liganded forms of Hb that contribute to the final spectra of the Hb mixture [22].

### 2.5 Large scale metHb synthesis

**Fig 1:**
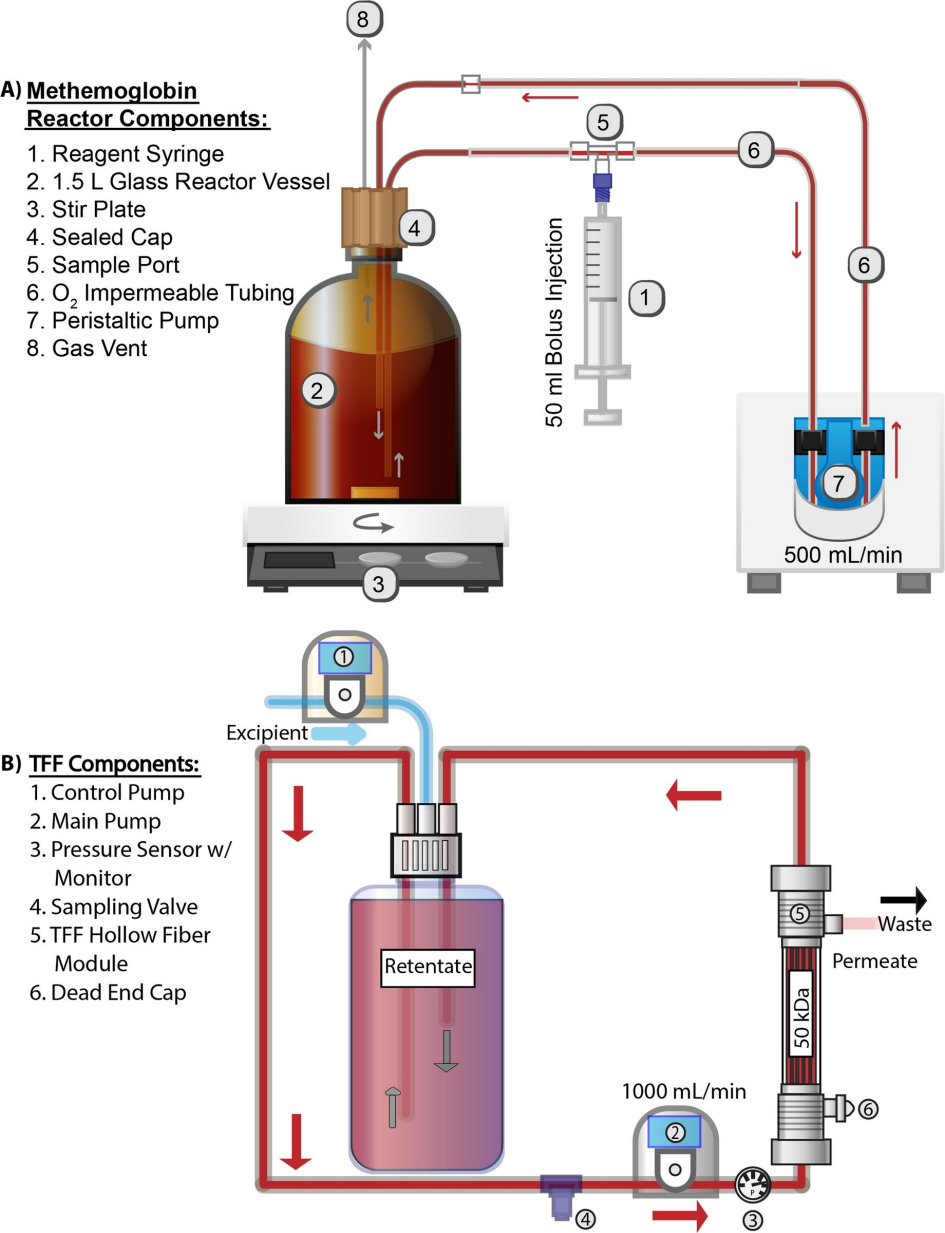
A) Schematic of reactor and fluid recirculation system used to synthesize metHb. B) Schematic of TFF system used to purify metHb.

Initially, 30 g of bHb/hHb was diluted in 1.5 L PBS (0.1 M, pH 7.4) and placed into an airtight, amber-tinted reactor vessel with continuous stirring as shown in **Fig 1A**. The reactor vessel coupled with a recirculation loop (500 mL/min) was placed in a fume hood at room temperature. After the recirculation loop was turned on, a bolus injection of 50 mL NaNO_2_ solution (0.013 mg/mL) was initiated through a 50 mL syringe attached to the sampling port at the inlet side of the reactor vessel. After two hours of reaction with constant stirring and recirculation, the metbHb/methHb solution was refrigerated at 4°C overnight.

### 2.6 MetHb clarification and purification

As shown in **Fig 1B**, both metbHb and methHb were initially transferred into a 2 L polypropylene bottle and concentrated down to 300 mL via TFF on a 50 kDa HF module. The metbHb/methHb solution was then transferred into a 1 L polypropylene bottle and subject to constant volume diafiltration with reductant-free modified lactated Ringer’s solution (115 mM NaCl, 4 mM KCl, 1.4 mM CaCl_2_.2H_2_O, 13 mM NaOH, 27 mM sodium lactate, pH 7.4). After 6 diafiltration cycles, the final metbHb/methHb product was concentrated to 270 mL at ~100 mg/mL and stored at -80°C.

### 2.7 Characterization of metHb

#### 2.7.1 Multi-species analysis during TFF

To evaluate the composition of metbHb/methHb mixtures as a function of TFF processing time, samples were collected every hour during TFF processing. The fraction of multiple Hb species at each time point including hemichrome, oxyHb, metHb, ferrylHb, metHb-NO_2_^-^, and NaNO_2_ was determined by spectra deconvolution using the Alchromy program.

#### 2.7.2 Haptoglobin-binding kinetics study

To evaluate haptoglobin (Hp) -Hb/metHb binding kinetics, a Hp mixture (0.25 μM, Hb tetramer binding basis) containing a mixture of Hp2-1 and Hp2-2 was purified from human Cohn Fraction IV [23]. The kinetics of Hp binding to Hb/metHb was measured as previously described in the literature [24]. The reaction between Hp and Hb/metHb was followed by stopped flow fluorescence spectrometry by excitation at 285 nm and monitoring the fluorescence emission at 310 nm [25, 26]. The pseudo first order Hp-Hb/metHb binding rate constant was calculated by fitting the fluorescence intensity to a monoexponential equation. The pseudo first-order rate constant was then used to determine the bimolecular rate constant via linear regression with the metHb/Hb concentration as the dependent variable.

#### 2.7.3 Hp-binding via size exclusion HPLC (SEC-HPLC) analysis

The molecular weight (MW) distribution of Hp-metHb mixtures was estimated via SEC-HPLC on an Acclaim SEC 1000 column (ThermoFisher Scientific, Waltham, MA). The binding capacity of Hp to Hb/metHb was analyzed by incubating a fixed concentration of Hp with Hb/metHb samples at different concentrations (1.25 μM, 2.5 μM, 5 μM, 10 μM, and 20 μM, Hb tetramer basis). All samples were analyzed via SEC-HPLC at a flow rate of 0.35 mL/min in the mobile phase (PB, 50 mM, pH 7.4). To determine the composition of Hp-metHb mixtures containing Hp-metHb complex, metHb tetramers (α_2_β_2_), dimers (αβ) and monomers (α/β), a deconvolution analysis was conducted based on the elution time at λ = 413 nm of each pure species.

#### 2.7.4 Total hemoglobin (Hb) and metHb level

The total Hb and metHb level of metbHb and methHb was measured using the cyanomethemoglobin assay as previously described in the literature [27].

#### 2.7.5 Purity and yield

The purity of both metbHb and methHb was defined as: $Purity\%=\frac{final\;mass\;of\;metHb}{final\;mass\;of\;total\;Hb\;species}$, which was determined from UV-visible spectral deconvolution analysis. The total mass of Hb was calculated based on the mass of each individual species including hemichrome, metHb, oxyHb, and metHb-${NO}_{2}^{-}$. The overall yield of metbHb and methHb was defined as: $Yield\%=\frac{final\;mass\;of\;metHb}{initial\;mass\;of\;Hb}$, which was determined by the cyanomethemoglobin assay described in section **2.7.4** [27].

#### 2.7.6 Storage stability analysis

The storage stability of bHb, hHb, metbHb and methHb was analyzed via analytical SEC-HPLC by incubating metHb samples at different concentrations (1.25 μM, 2.5 μM, 5 μM, 10 μM, and 20 μM, heme basis) with 0.1 M PBS solution (pH 7.4) at 4°C for one week. To determine the composition of denatured metHb mixtures containing metHb tetramers, dimers and monomers, a deconvolution analysis was performed based on the elution time of each pure species. To quantitively evaluate the dimerization equilibrium of Hb, the tetramer-dimer dissociation constant (K_d_) was derived as follows:

$$\mathrm{Hb}\;\mathrm{tetramer}\overset{K_{d}}{↔}\mathrm{Hb}\;\mathrm{dimer}+\mathrm{Hb}\;\mathrm{dimer}$$ (1)

$$K_{d}=\frac{{\left[D\right]}^{2}}{\left[T\right]}=\frac{{\left[2([H]-[T])\right]}^{2}}{\left[T\right]}=\frac{{4[H][1-T\%]}^{2}}{\left[T]/[H\right]}=\frac{{4[H][1-T\%]}^{2}}{T\%}$$ (2)

$${log}_{10}(1/K_{d})={log}_{10}\left(\frac{T\%}{{4[1-T\%]}^{2}}\right)-{log}_{10}[H]$$ (3)

where [H] is the initial concentration of Hb/metHb tetramer, [T] is the concentration of Hb/metHb tetramers, [D] is the concentration of Hb/metHb αβdimers and T% is defined as the ratio [T] / [H]. Thus, a plot of ${log}_{10}\left(\frac{T\%}{{4[1-T\%]}^{2}}\right)$ with respect to *log*_10_[*H*] yielded an intercept of *log*_10_(1/*K_d_*) as shown in **Eq** **3** [28, 29].

#### 2.7.7 Statistical analysis

A t-test was performed to study the variances among different groups of data, and a p-value (< 0.05) demonstrates a significant difference. All data were reported with the standard deviation, and the R-squared (R^2^) value was used to evaluate the performance of the regression model.

## 3 Results and discussion

### 3.1 Optimal molar ratio of Hb (heme basis) to oxidization agent

**Fig 2:**
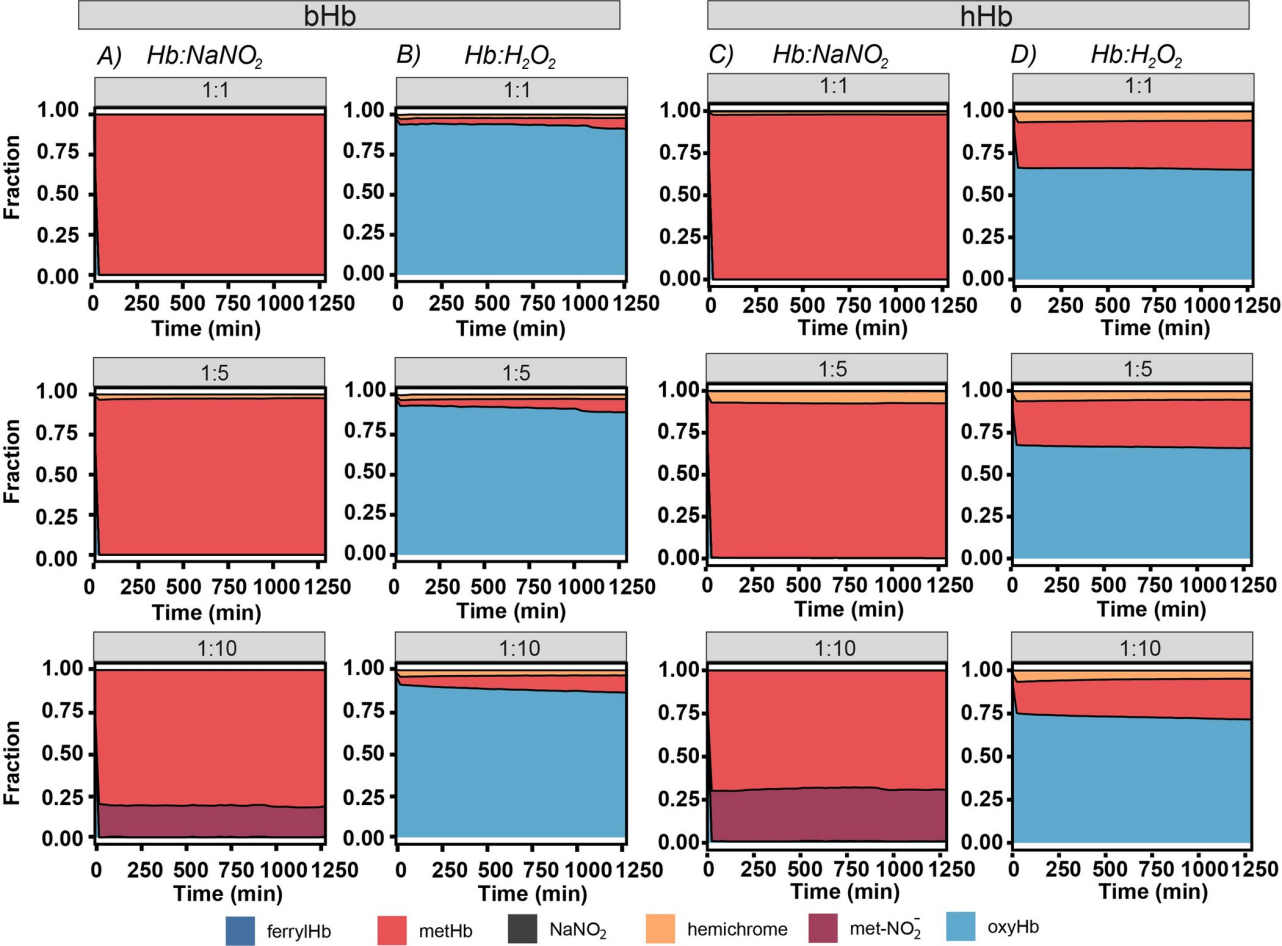
Oxidization of Hb at varying Hb:oxidization agent molar ratio (heme basis, 1:1, 1:5, and 1:10). The oxidation of 60 μM Hb (heme basis) with either NaNO_2_ or H_2_O_2_ was monitored via UV-visible spectrometry in 0.1 M PBS pH 7.4 at room temperature for 21 h. Since multiple Hb species were observed, a deconvolution program was used to determine the composition of each Hb species within the reaction mixture at each time point.

In this initial study, two different oxidization agents (NaNO_2_ and H_2_O_2_) were tested within a 21 hour window to determine the optimal molar ratio of Hb:oxidization agent, which led to the highest yield of metHb. The optimal oxidization agent and molar ratio of Hb:oxidization agent was then used for subsequent large scale synthesis of metHb. **Fig 2** shows the composition of metHb mixtures prepared by reacting three different molar ratios of Hb:oxidization agent (1:1, 1:5, and 1:10). In **Fig 2A and 2C**, the oxidation of both bHb/hHb at 1:1 and 1:5 molar ratios of Hb:NaNO_2_ displayed rapid conversion of Hb to metHb, which is consistent with previously reported studies [20]. Approximately ~95% of oxyhemoglobin (oxyHb) was converted to metHb within the first 30 mins of the reaction with negligible formation of hemichrome, suggesting high conversion to metHb with minimal side products.

For NaNO_2_ oxidation of both bHb and hHb at a molar ratio of 1:5 in **Fig 2A and 2C**, a slightly higher fraction of hemichrome was produced in comparison to a molar ratio of 1:1, which can be attributed to the excess NaNO_2_ in solution. ${NO}_{2}^{-}$ most likely converts the high-spin metHb to low-spin hemichrome due to ligation of the distal histidine residue in the heme pocket [30–32]. At a molar ratio of 1:10, it is interesting to note that adding more NaNO_2_ did not lead to substantial formation of hemichrome, but instead led to the formation of a large fraction of metHb-${NO}_{2}^{-}$. This can be explained by the dramatic pH increase in solution due to excess NaNO_2_, which impeded hemichrome formation and facilitated ${NO}_{2}^{-}$ binding to metHb [33]. Oxidation at molar ratios of 1:1 and 1:5 yielded relatively high conversion of Hb to metHb (97.6% and 97.8% for bHb and hHb respectively) and negligible side products in comparison to that at a molar ratio of 1:10 (~87.8% and 70.1% for bHb and hHb respectively). For Hb:H_2_O_2_ molar ratios of 1:1, 1:5, and 1:10, the final concentration of metHb was found to be 6.4%, 7.9% and 10.6% for bHb (**Fig 2B**), respectively. In **Fig 2D**, relatively higher conversions (29.1%, 28.7% and 23.7%) of hHb to methHb at various Hb:H_2_O_2_ molar ratios (1:1, 1:5, and 1:10) were observed due to the initially higher metHb level of hHb in comparison to bHb. Unfortunately, the final yield of metHb using H_2_O_2_ was still significantly lower than that using NaNO_2_ (t-test, p < 0.05), indicating that H_2_O_2_ exhibited a weaker ability to convert Hb into metHb in comparison to NaNO_2_. Additionally, H_2_O_2_ could induce chemical modification of the Hb protein structure, which might interfere with the binding between metHb and Hp [34]. Taken together, H_2_O_2_ is not an ideal oxidization agent for the synthesis of metHb. Thus, in this study, NaNO_2_ was used as the oxidization agent for scaleup synthesis of metHb. To further reduce the reaction time in the large scale metHb synthesis scheme, a Hb:NaNO_2_ molar ratio of 1:5 was used for both metbHb and methHb synthesis.

### 3.2 Molecular weight

**Fig 3:**
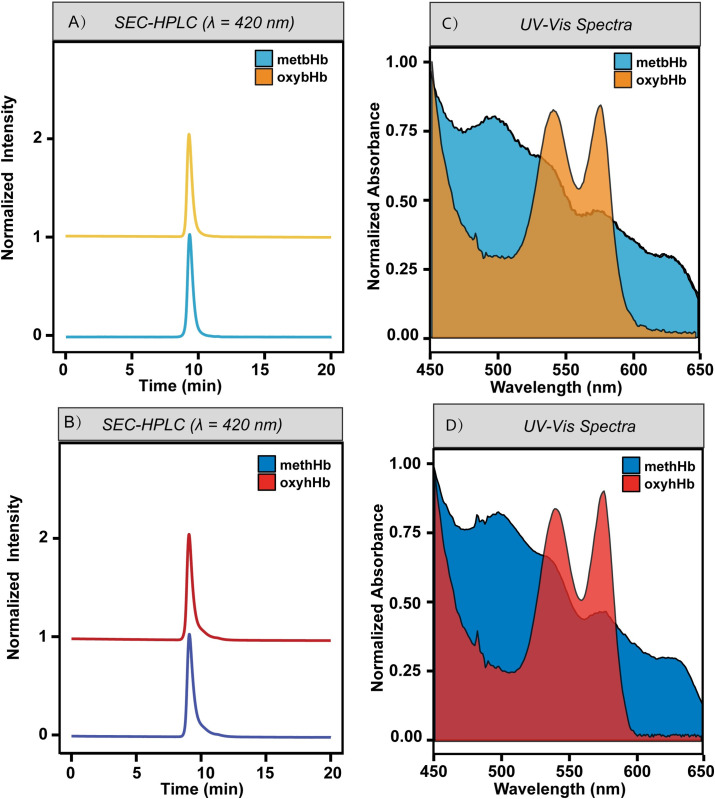


**Fig 3A and 3B** show the SEC-HPLC chromatogram of bovine/human oxyHb (oxybHb/oxyhHb), and freshly synthesized metHb (1 mg/mL, heme basis), which both eluted at the same time (9.29 mins). The molecular weight (MW) of oxybHb/oxyhHb and metbHb/methHb was estimated to be ~ 64 kDa, which is consistent with the theoretical MW [24]. Additionally, a single sharp peak was observed for both metbHb and methHb, indicating the high purity of the final product. The decreasing intensity of metHb compared to Hb at the same concentration was observed for both metbHb and methHb, which can be attributed to the left-shifted Soret peak of metbHb from 413 nm to 405 nm [35]. To further confirm the presence of metbHb/methHb, the full UV-visible spectra of metbHb and methHb were extracted from the SEC-HPLC chromatogram and displayed in **Fig 3C and 3D**. Four distinct peaks (505 nm, 540 nm, 575 nm and 629 nm) were observed, which aligns well with the literature spectra of metHb [36].

### 3.3 Composition of Hb species during TFF

**Fig 4:**
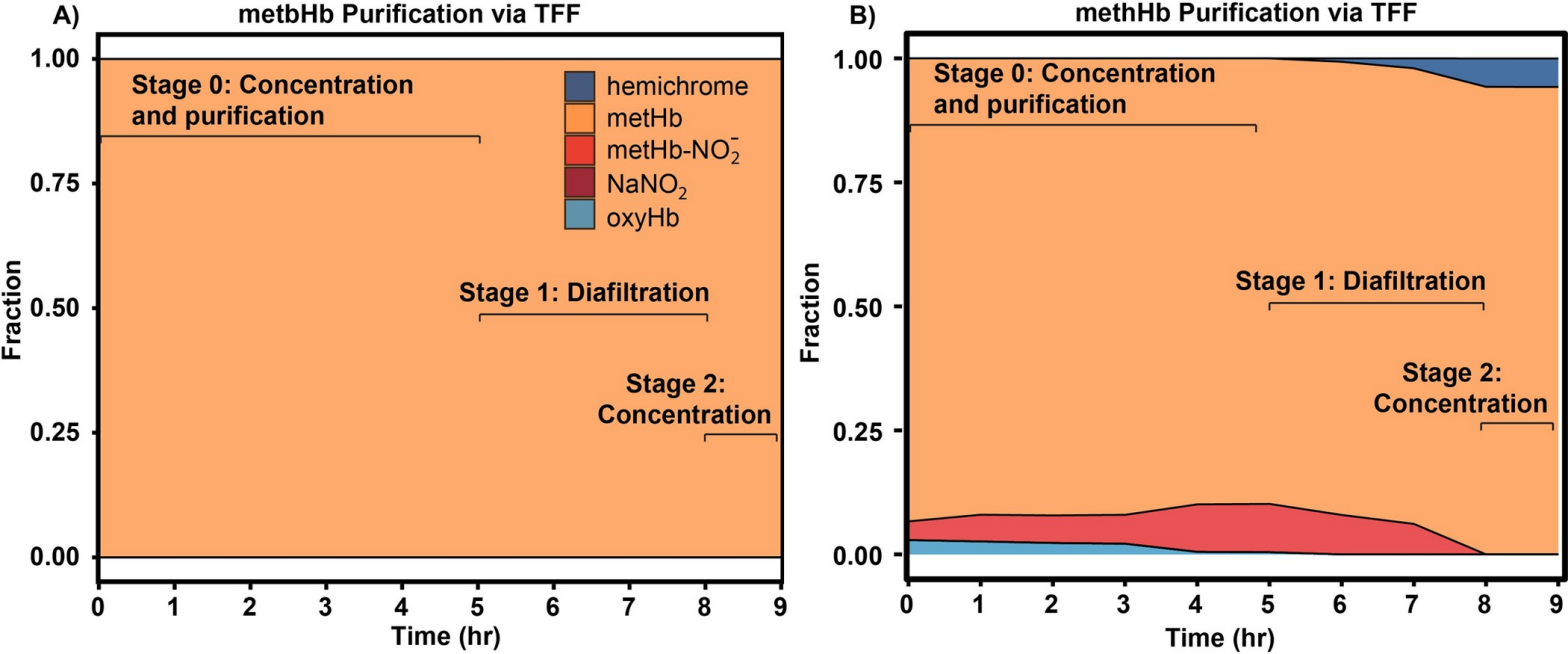
A) Composition of bovine metHb (metbHb, 1.5 L reactor system) and (B) human metHb (methHb, 1.5 L reactor system) during TFF processing. Stage 0: Both metbHb and methHb were initially transferred into a 2 L polypropylene bottle and concentrated to 500 mL (total volume). Stage 1: The solution was then subject to constant volume diafiltration on a 50 kDa HF module within a 500 mL bottle. Stage 3: The solution at the end of Stage 1 was further concentrated to ~100 mg/mL and stored at -80°C.

To evaluate the composition of metbHb/methHb mixtures as a function of TFF processing time after large scale synthesis, samples were collected every hour during the TFF process. The fraction of multiple Hb species including hemichrome, oxyHb, metHb, ferrylHb, metHb-NO_2_^-^, and NaNO_2_ was determined via spectra deconvolution using the Alchromy program. In **Fig 4A**, a mixture consisting of almost pure metHb can be observed at all stages during the TFF metbHb purification process, which is consistent with results from the small scale oxidization agent experiments. For TFF methHb purification in **Fig 4B**, the composition changed at different stages during the purification process. At the beginning of stage 0, a mixture of 93.4% metHb, 2.8% oxyHb and 3.8% metHb-NO_2_^-^ was observed which could be due to the initially higher metHb level of hHb (~5%) in comparison to bHb (0.9%). The initially high metHb level of hHb is attributed to the fact that the hHb was purified from expired human RBCs, which possess a higher starting metHb level versus hHb derived from fresh human RBCs or RBCs used before the expiration date. At the end of stage 0, all oxyHb was converted to metHb-NO_2_^-^ likely due to the presence of residual NaNO_2_ in solution. After diafiltration was initiated (Stage 1), a dramatic decrease in the fraction of metHb-NO_2_^-^ was observed at the end of the diafiltration process. This can be explained by the increased conversion of metHb-NO_2_^-^ into metHb by removal of the residual NO_2_^-^ via diafiltration on the 50 kDa HF module, demonstrating the importance of the diafiltration process using TFF in removal of excess reagents. After the last volume exchange, spectral deconvolution analysis was performed on the final material, which led to a purity of 99.7 ± 0.2% and 87.4 ± 4.2% for metbHb and methHb, respectively. It was found that the final methHb material contained a higher amount of hemichrome and metHb-NO_2_^-^ compared to the synthesized metbHb. MethHb also possessed a lower yield (77.4 ± 3.9%) compared to methHb (89.3 ± 0.8%), which could be a consequence of the higher starting metHb level for hHb compared to bHb.

### 3.4 MetHb-Hp binding kinetics

The human Hp used in this study consisted of a mixture of phenotypes Hp2-1 and Hp2-2, which functions as a Hb scavenger to inhibit oxidation-mediated reactions elicited by the iron atom in Hb. Hp binds to Hb to form the Hp-Hb complex and removes it from the systemic circulation via CD163 receptor mediated endocytosis into macrophages and monocytes [37]. To determine the potential for metbHb to be cleared via CD163 mediated endocytosis, the ligand-binding kinetics of Hp with bHb/metbHb was monitored by rapidly mixing Hp with bHb/metbHb at various concentrations.

**Fig 5:**
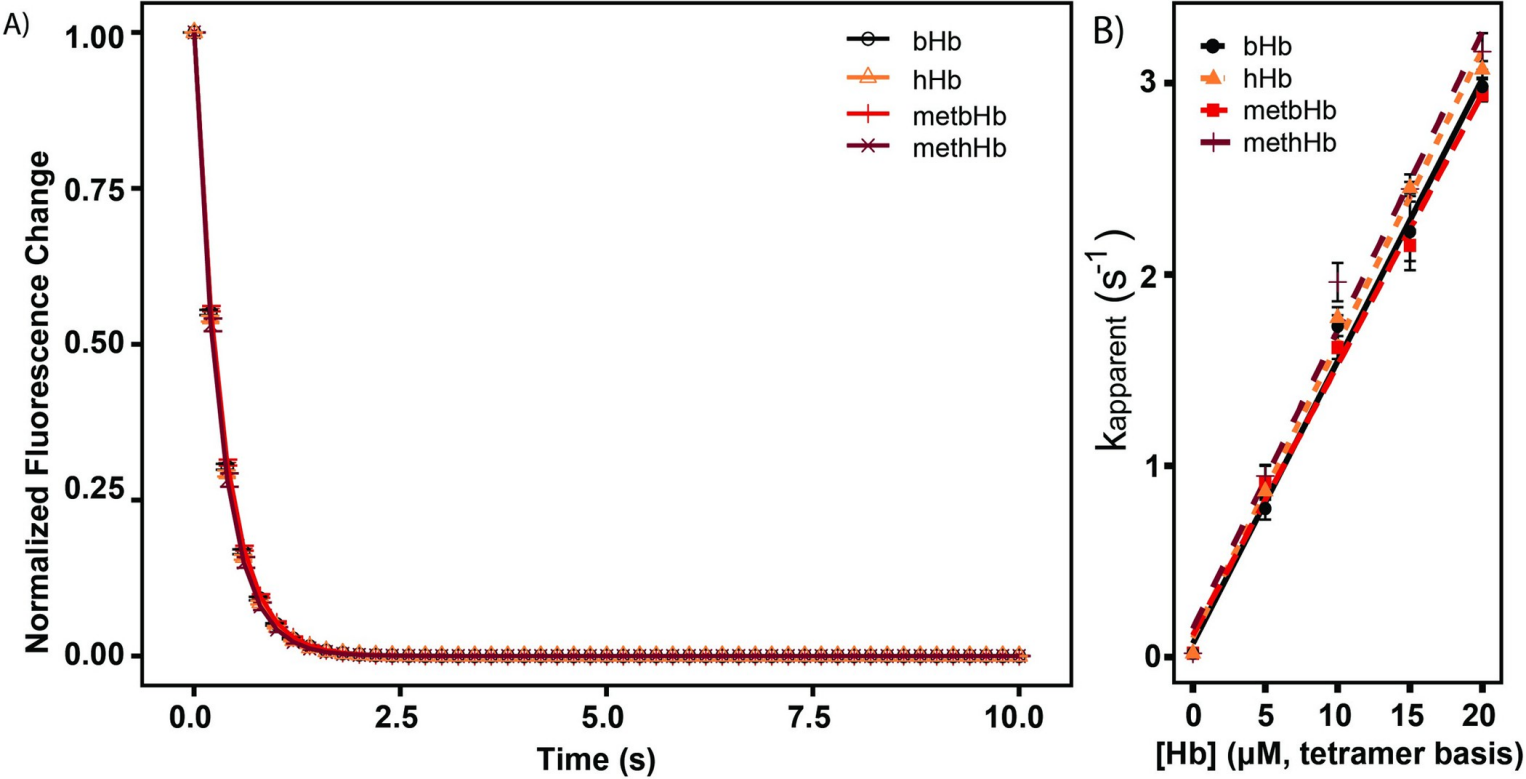
**A) Pseudo first order Hp binding kinetics of Hb/metHb.** Excess Hb/metHb was rapidly mixed with Hp (0.25 μM on a Hb tetramer binding basis). The normalized fluorescence changes were fit to a monoexponential equation to regress the pseudo first order Hp binding rate constant. **B) Dependence of the pseudo first order Hp binding rate constant as a function of Hb/metHb concentration.** The second order Hp binding rate constant was obtained by performing a linear fit of the pseudo first order Hp binding rate constant to the Hb/metHb concentration. (The error bars represent the standard deviation from 3 replicates).

In **Fig 5A**, the kinetics of Hp-bHb/metHb binding is shown, where metbHb was found to quench a similar number of Hb-binding sites in Hp in comparison to non-oxidized bHb. To determine the pseudo first order binding rate constants, kinetic traces were fit to a monoexponential equation (**Fig 5B)**. To calculate the 2^nd^ order (bHb/metbHb)-Hp binding rate constant (k_Hp-Hb_), a linear fit to the data in **Fig 5B** to determine the slope of the pseudo first order reaction rate constant as a function of metbHb/bHb concentration. Overall, k_Hp-Hb_ of metbHb (0.140 ± 0.003 μM^−1^ s^−1^) exhibited no significant difference compared to unmodified bHb (0.147 ± 0.006 μM^−1^ s^−1^), methHb (0.156 ± 0.003 μM^−1^ s^−1^), and unmodified hHb (0.154 ± 0.003 μM^−1^ s^−1^).

### 3.5 MetHb-Hp binding via SEC-HPLC analysis

**Fig 6:**
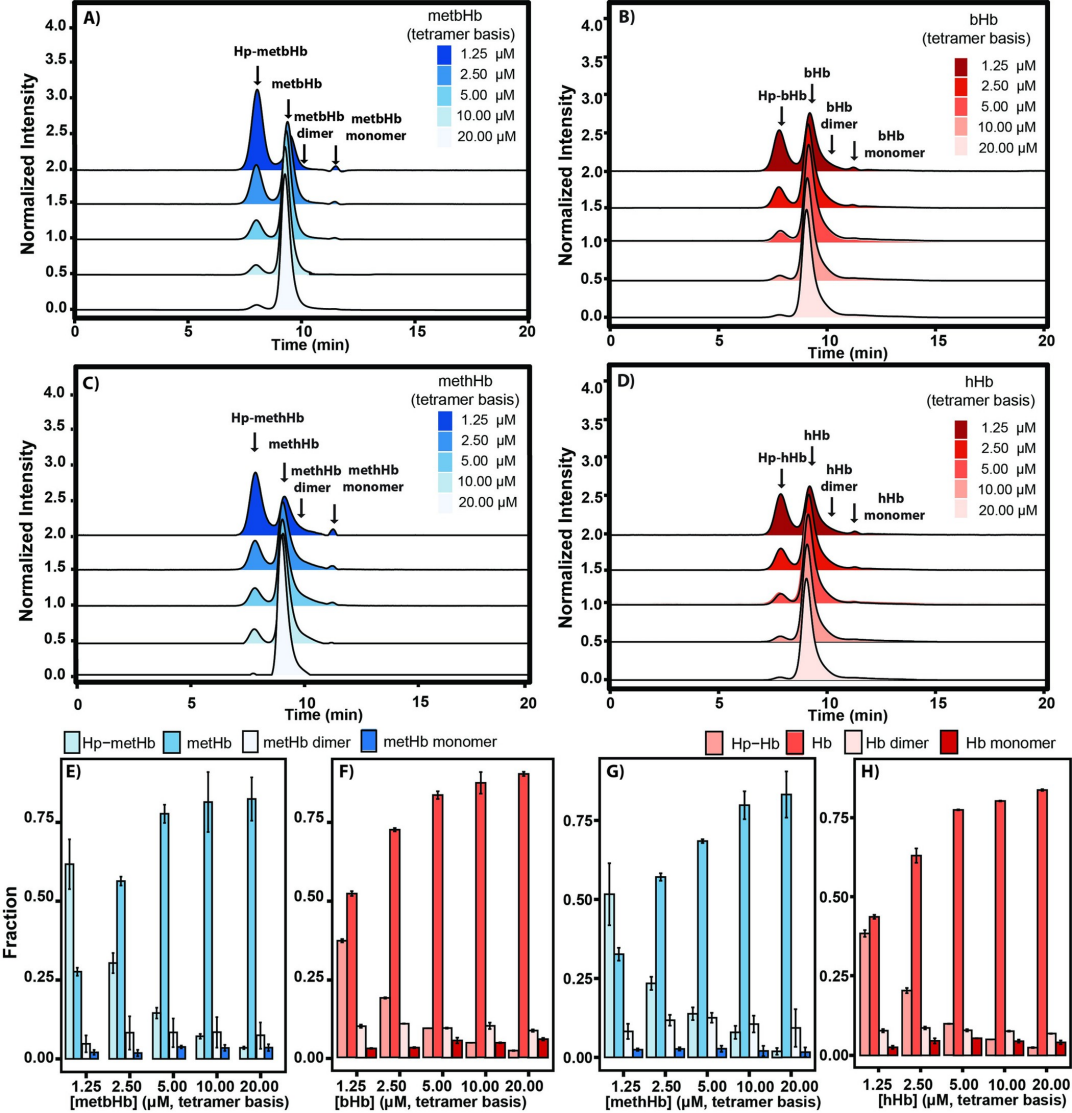
SEC-HPLC of Hp-metHb/Hb complexes as a function of metHb/Hb concentration. **Normalized SEC-HPLC chromatograms of: (A) Hp-metbHb, (B) Hp-bHb, (C) Hp-methHb, and (D) Hp-hHb mixtures.** Chromatograms were monitored at an absorbance of λ = 413 nm normalized against the total area under the curve. Hp (0.25 μM on a Hb tetramer binding basis) was rapidly mixed with excess metHb/Hb. **Composition of: (E) Hp-metbHb, (F) Hp-bHb), (G) Hp-methHb, and (H) Hp-hHb mixtures which include the Hp-metHb/Hb complex, metHb/Hb tetramer (α**_**2**_**β**_**2**_**), metHb/Hb dimer (αβ) and monomer (α/β) as a function of metHb/Hb concentration.** (The error bars represent the standard deviation from duplicate experiments).

As shown in **Fig 6A and 6C**, the molar fraction of Hp binding to metbHb/methHb was measured via analytical SEC-HPLC. The Hp mixture purified in our lab contained Hp 2–1 (~200 kDa) and Hp 2–2 (~400 kDa). To analyze the Hp−metHb complex, metbHb/methHb at different molar concentrations were initially mixed with excess Hp (2:1 Hp:metHb) as previously described in the literature [38]. In comparison to Hp binding of native Hb, both bHb and hHb were prepared using the same protocol. A deconvolution program was then used to determine the molar fraction of each species including Hp-metHb complexes, metHb tetramers (α_2_β_2_), metHb dimers (αβ), and metHb monomers (α/β globin). The elution time of Hp-metHb was found to be ∼8.02 min, which corresponds to a MW of ~400−500 kDa. It is evident that decreasing the molar concentration of metHb resulted in increased binding of Hp to metHb (from 0.78 μM to 0.87 μM), as anticipated. Hp binding facilitates dimerization of both metbHb and methHb, which led to a substantial reduction in the amount of tetrameric metbHb (from 82.4 ± 6.9% to 27.6 ± 1.3%) and methHb (from 83.2 ± 7.3% to 32.6 ± 2.0%) in solution (**Fig 6A and 6C**). Interestingly, both metbHb and methHb exhibited higher Hp binding capacity in comparison to bHb and hHb (**Fig 6B and 6D**) at relatively low concentrations (<5 μM, heme basis). This can be explained by the facilitated dimerization elicited by reducing the Hb concentration in solution.

In **Fig 6E and 6G**, no significant difference (t-test, p > 0.05) was observed between the metbHb/methHb fraction at concentrations of 5 μM and 10 μM, and 10 μM and 20 μM (Hb tetramer basis), indicating that the Hp binding equilibrium was not significantly affected by molar concentrations of Hb > 5 μM. In the range 0–5 μM Hb, Hp-metHb binding was increased by lowering the metHb concentration, which was also observed for bHb/hHb in **Fig 6F and 6H**. Taken together, this indicates that the synthesized metHb can be potentially cleared from the systemic circulation via binding to Hp in a similar manner to Hb. The difference between the binding equilibrium of Hp-metHb and Hp-Hb can only be observed at metHb/Hb molar concentrations < 5 μM.

### 3.6 Storage stability

**Fig 7:**
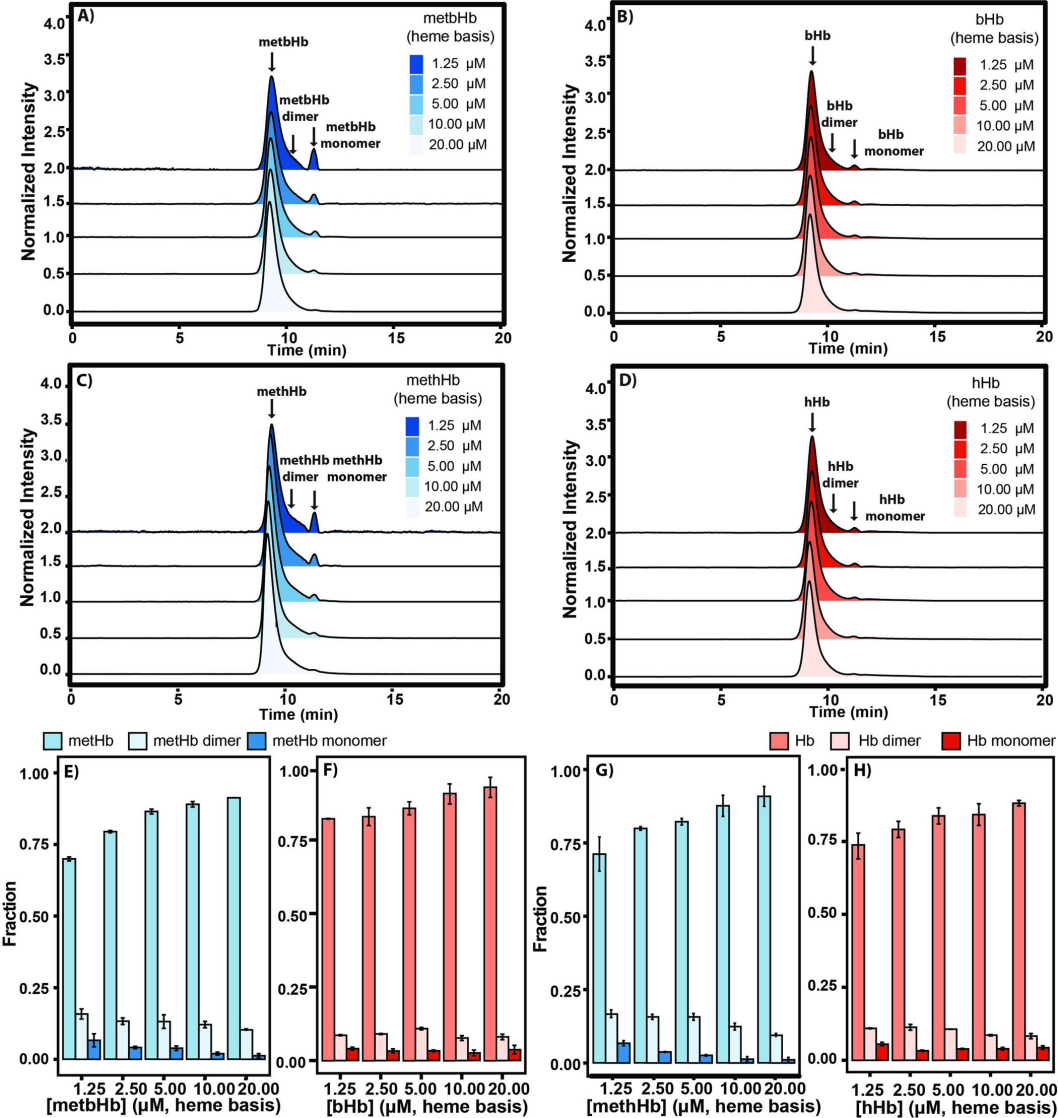
SEC-HPLC of metHb/Hb as a function of metHb/Hb concentration to monitor metHb/Hb dissociation after 1 week of storage at 4°C. **Normalized intensity SEC-HPLC chromatograms of (A) metbHb, (B) bHb, (C) methHb, and (D) hHb.** Chromatograms were monitored at an absorbance of λ = 413 nm normalized against the total area under the curve. **Composition of (E) metbHb, (F) bHb, (G) methHb, and (H) hHb species** including the metHb/Hb tetramer (α_2_β_2_), metHb/Hb dimer (αβ) and monomer (α/β) as a function of metHb/Hb concentration. (The error bars represent the standard deviation from duplicate experiments).

In **Fig 7A and 7C**, the effect of the molar concentration of metbHb/methHb on its ability to dimerize into αβ dimers was examined by analytical SEC-HPLC. In this study, a serial dilution was performed to prepare metHb solutions at molar concentrations of 1.25 μM, 2.5 μM, 5 μM, 10 μM, and 20 μM (heme basis) after incubation in PBS (0.1 M, pH 7.4) at 4°C for one week. In comparison to the stability of metbHb/methHb, native bHb/hHb was prepared following the same protocol (**Fig 7B and 7D**). To determine the molar fraction of each metHb species including metHb tetramers (α_2_β_2_), metHb dimers (αβ), and metHb monomers (α/β globin), a deconvolution analysis was conducted as previously described. An increasing molar fraction of metHb dimers (from 9.5 ± 0.3% to 15.8 ± 1.7%) and monomers (from 1.2 ± 0.7% to 6.6 ± 2.2%) was observed when lowering the molar concentration of metbHb from 20 μM to 1.25 μM (heme basis). Akin to metbHb, the fraction of methHb dimers increased from 9.4 ± 0.6% to 13.7 ± 1.3%, and from 1.1 ± 0.8% to 6.7 ± 0.9% for metHb monomers, upon dilution. In comparison to metbHb/methHb, slight Hb dissociation was observed for native bHb/hHb upon dilution (**Fig 7B and 7D**).

In **Fig 7E and 7G**, a negative correlation was observed between the fraction of dissociated metHb species (dimers and monomers) and the molar concentration of metbHb/methHb, indicating that the metbHb/methHb dissociation process is facilitated by reducing the molar concentration of metHb in solution. Additionally, both metbHb/methHb dimers and monomers were lower than 18% even at the extremely low concentration (1.25 μM, heme basis) after storage at 4°C for one week, demonstrating the high stability of the synthesized metHb.

In **Fig 7F and 7H**, less variation was observed among all groups of the bHb/hHb tetramer at various concentrations in comparison to methHb/methHb, indicating that native Hb is more stable than metHb. The oxidation of heme from the ferrous state (Fe^2+^) to the ferric state (Fe^3+^) accelerates the conversion of tetrameric metHb into dimeric metHb. In general, both metbHb and methHb possessed greater than 87% of metHb tetramers at a concentration of 20 μM (heme basis). Thus, negligible tetramer dissociation can be anticipated when used *in vivo* at relatively high protein concentrations.

**Fig 8:**
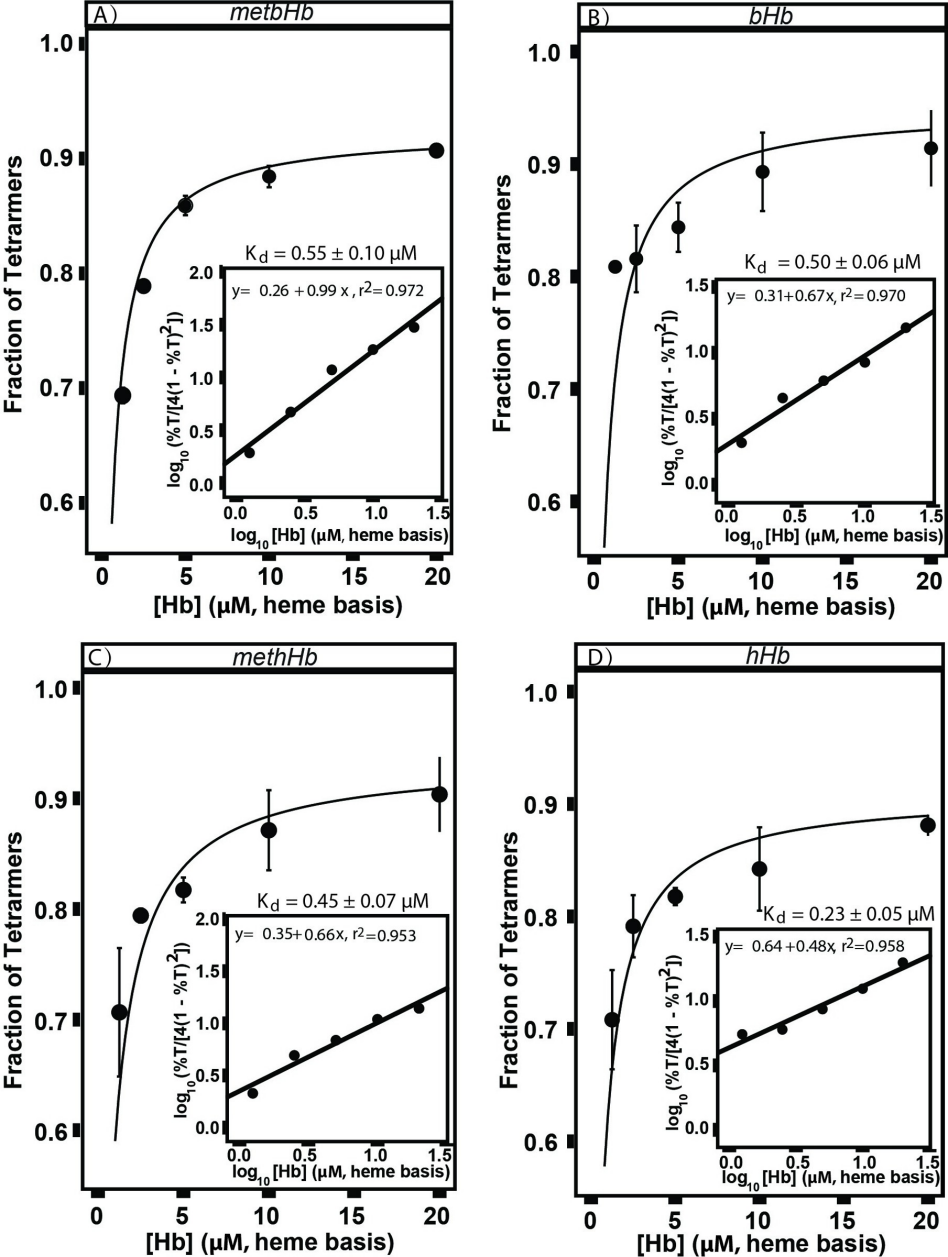
Equilibria between dimers and tetramers of (A) metbHb, (B) bHb, (C) methHb, (D) hHb. The tetramer-dimer dissociation constant (K_d_) shown in each figure inset was calculated as described in the text. All samples were incubated in PBS (0.1 M, pH 7.4) and analyzed on a Thermo Scientific Dionex UltiMate 3000 UHPLC/HPLC system coupled with an Acclaim SEC-1000 column. Deconvolution analysis was then performed on the chromatogram of all samples to yield the fraction of Hb tetramers. (The error bars represent the standard deviation from 3 replicates; the regression performance was evaluated with the R-squared (R^2^) value).

In **Fig 8A and 8C**, the fraction of metbHb/methHb tetramers was plotted as a function of metbHb/mehHb concentration, which yielded a hyperbolic profile. To determine the tetramer-dimer dissociation constant (K_d_), a linear transformation was performed on the fraction of metbHb/methHb tetramers by using **Eq 3**, whose intercept yielded log(1/K_d_). The K_d_ of metbHb (0.55 ± 0.10 μM) and methHb (0.45 ± 0.07 μM) can be further derived from the intercept value, which are comparable to previously reported values in the literature [39, 40]. In **Fig 8B and 8D**, it was found that native bHb and hHb possessed a K_d_ value of 0.50 ± 0.06 μM and 0.23 ± 0.05 μM, respectively, when incubated in PBS (0.1 M, pH 7.4) at 4°C. Although the K_d_ of metbHb/methHb seems to be slightly higher than native bHb/hHb, it is still on the same order of magnitude as the values for native Hb. In general, these results show that the conversion of heme from the ferrous state (Fe^2+^) to ferric state (Fe^3+^) has little effect on tetramer dissociation in the concentration range 1.25–20 μM, (heme basis) in PBS solution (pH 7.40).

## 4 Conclusion

In this study, we identified the optimal molar ratio of Hb:NaNO_2_ to synthesize metHb with high yield and purity using a batch reactor and TFF separation train. Additionally, no significant difference was found between the second order binding rate constant of Hp to metHb/Hb. Previous studies observed that elevated levels of nitrite in blood can oxidize the Hb inside RBCs, leading to methemoglobinemia. In this study, a negligible amount of nitrite was observed within the final metHb product, indicating the potential safety of using metHb as a suitable control material in small animal models. Future work might include evaluation of the safety and stability of this material *in vivo*. Taken together, both the metbHb and methHb synthesized in this study were stable at 4°C in PBS (0.1 M, pH 7.4) for at least one week. The Hp-binding kinetics and equilibria of metbHb/methHb were comparable at metHb concentrations ranging from 5–20 μM (Hb tetramer basis). The difference between the Hp-binding equilibria of metHb and Hb can only be observed at extremely low concentrations (< 5 μM, Hb tetramer basis). The biophysical properties of the metHb synthesized in this study demonstrate the potential of using it as a non-O_2_ carrying control material in studies of cell-free hemoglobin solutions.

## Supporting information

S1 File Storage and kinetics study. SEC-HPLC chromatograms, far UV CD spectra, and ligand-binding properties of bHb, hHb, metbHb, and methHb.
(DOCX)

S2 File Minimal dataset. (XLSX)

## Abbreviations

DI water: Deionized water
Hb: Hemoglobin
HF: Hollow fiber
hHb: Human Hb
bHb: Bovine Hb
metbHb: Bovine methemoglobin
methHb: Human methemoglobin
Hp: Haptoglobin
MetHb: Methemoglobin
MW: Molecular weight
*N*: Cooperativity coefficient
NO: Nitric oxide
PB: Phosphate buffer
PBS: Phosphate buffered saline
RBC: Red blood cell
SEC: Size-exclusion chromatography
HPLC: High performance liquid chromatography
TFF: Tangential flow filtration
MAP: Mean arterial pressure
