# Supplementary material for: Scalable manufacturing platform for the production of methemoglobin as a non-oxygen carrying control material in studies of cell-free hemoglobin solutions
Source: PLoS One. 2022 Feb 16;17(2):e0263782. doi: 10.1371/journal.pone.0263782 (PMC8849478; doi:10.1371/journal.pone.0263782)
Supplement: S1 File — SEC-HPLC chromatograms, far UV CD spectra, and ligand-binding properties of bHb, hHb, metbHb, and methHb. (DOCX) [file pone.0263782.s001.docx]

**Storage and kinetics study**


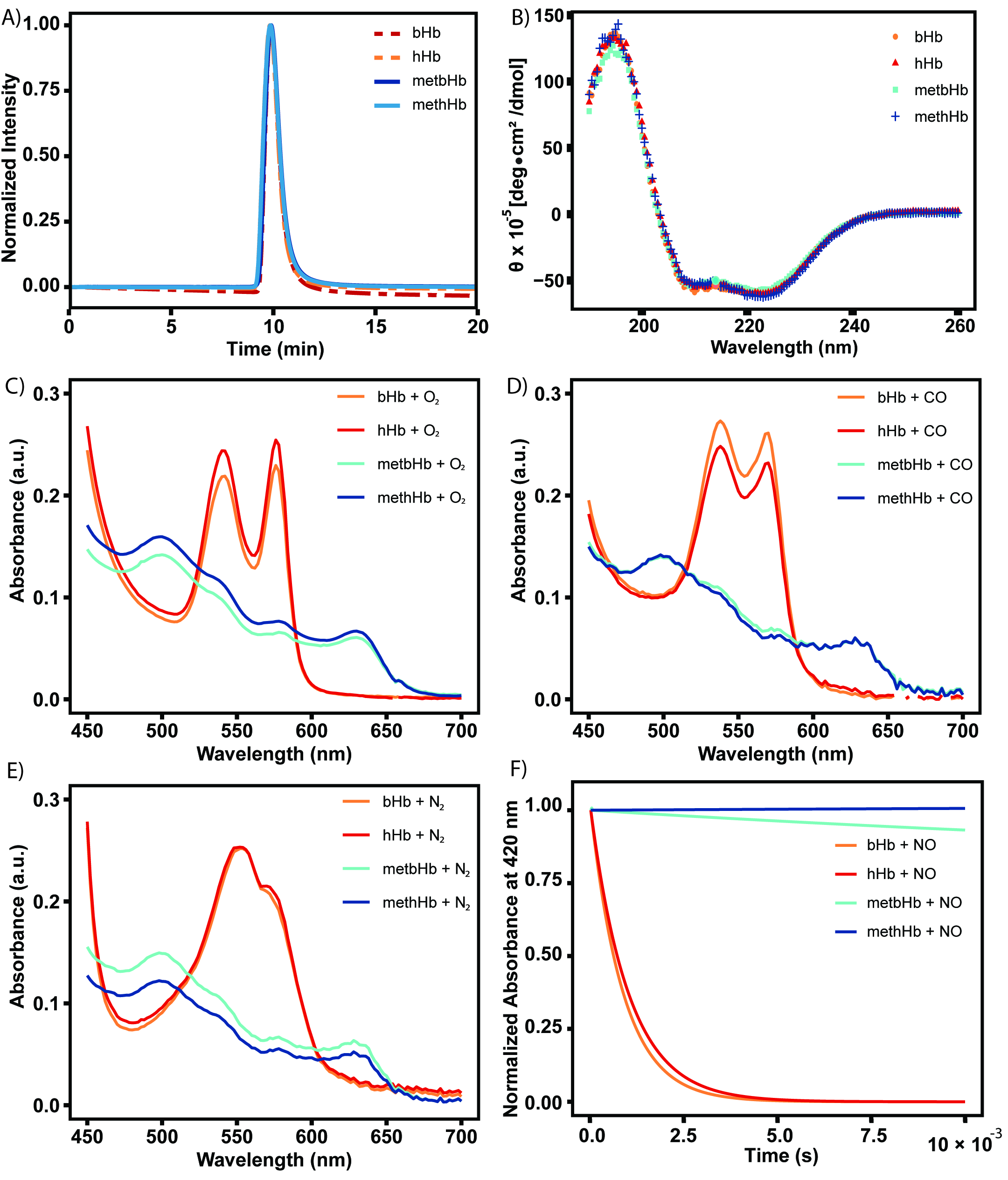


**Figure S1. (A) SEC-HPLC chromatograms of bHb, hHb, metbHb, and methHb after 1 year storage at -80 °C.** SEC-HPLC was performed using a Thermo Scientific Dionex UltiMate 3000 UHPLC/HPLC system coupled with an Acclaim SEC-1000 column. **(B) Far UV CD spectra (from 190 to 260 nm) of bHb, hHb, metbHb, and methHb**. The CD spectra was evaluated in a quartz cuvette (1 mm path length) and analyzed using the JASCO J-815 CD (JASCO, Easton, MD) spectrometer [1]. **(C) UV-visible spectra of bHb, hHb, metbHb, and methHb after 1 year storage at -80 °C in the oxygenated state.** The absorbance spectra was recorded in 0.1 M PBS pH 7.4 at room temperature. The spectra was then used for deconvolution analysis (**Table S1**). **(D) UV-visible spectra of bHb, hHb, metbHb, and methHb equilibrated with CO and (E) N_2_** at room temperature in PBS (0.1 M, pH 7.4). **(F)** **Stopped-flow kinetics for the NO binding reaction** at 420 nm with oxygenated bHb, hHb, metbHb, and methHb. The analysis was performed in PBS (0.1 M, pH 7.4) saturated with NO (25 μM).

**Table S1. Deconvolution analysis of metbHb and methHb after 1 year storage at -80 °C equilibrated with O_2_.**

| **MetbHb** | | | |
| --- | --- | --- | --- |
| **Species** | **Percent (%)** | **Standard error*** | **R^2^** |
| **Hemichrome** | 0.00 | 0.005738 | 0.99501 |
| **OxyHb** | 0.00 | 0.001608 |  |
| **metHb** | 100.00 | 0.003391 |  |
| **metHb-**$\mathbf{NO}_{\mathbf{2}}^{\mathbf{-}}$ | 0.00 | 0.00866 |  |
| **MethHb** | | | |
| **Species** | **Percent (%)** | **Standard error*** | **R^2^** |
| **Hemichrome** | 0.00 | 0.004144 | 0.99805 |
| **OxyHb** | 0.00 | 0.001161 |  |
| **metHb** | 100.00 | 0.002448 |  |
| **metHb-**$\mathbf{NO}_{\mathbf{2}}^{\mathbf{-}}$ | 0.00 | 0.006253 |  |

The final metbHb/methHb product was stored at -80°C for ~ 1 year, and compared to bHb and hHb stored under the same conditions. From the SEC-HPLC results in **Figure S1A**, both metbHb and methHb exhibited similar elution time in comparison to bHb and hHb after storage at -80 °C for ~1 year, indicating negligible aggregation during storage. Circular dichroism (CD) spectroscopy analysis of bHb, hHb, metbHb, and methHb are shown in **Figure S1B**. The CD spectra of metbHb and methHb overlapped with bHb and hHb after 1 year storage at -80°C, indicating that the secondary structure of metbHb and methHb remained unchanged after oxidation of bHb and hHb, respectively. Additionally, the oxidation state of the heme was evaluated using UV-visible spectroscopy after 1 year storage at -80°C. The characteristic peaks of metbHb and methHb (λ = 505 nm, 540 nm, 575 nm and 629 nm) remained unshifted (**Figure S1C**). The spectral deconvolution analysis which included multiple Hb species were performed on the metHb samples stored after 1 year. There was no drastic change in the composition of either metbHb or methHb as shown in the **Table S1.** We also measured the ligand-binding properties of bHb, hHb, metbHb, and methHb after 1 year storage at -80°C including O_2_ , CO, and NO binding in **Figure S1C, D, and F**. Both metbHb and methHb exhibited negligible changes after equilibration with O_2_, N_2_ and CO. In **Figure S1F**, The NO binding rate of metbHb (39 s^-1^) and methHb (35 s^-1^) was drastically slower than that of native bHb (983 s^-1^) and hHb (1139 s^-1^). The rate constant of hHb was comparable to values in the literature [2], which led to the overall conclusion that there was negligible effect of the freeze thaw process on the biophysical characteristics of the synthesized metbHb/methHb.

**Reference**

1. Zhang N, Jia Y, Chen G, Cabrales P, Palmer AF. Biophysical properties and oxygenation potential of high-molecular-weight glutaraldehyde-polymerized human hemoglobins maintained in the tense and relaxed quaternary states. Tissue Eng - Part A. 2011;17: 927–940. doi:10.1089/ten.tea.2010.0353

2. Belcher DA, Banerjee U, Baehr CM, Richardson KE, Cabrales P, Berthiaume F, et al. Mixtures of tense and relaxed state polymerized human hemoglobin regulate oxygen affinity and tissue construct oxygenation. PLoS One. 2017;12. doi:10.1371/journal.pone.0185988
